# Extraction and Thermal Stability of Chlorogenic Acid from Green Coffee and Yerba Mate in Food-Based Solvents

**DOI:** 10.1021/acsomega.6c04214

**Published:** 2026-08-10

**Authors:** Monique Martins Strieder, Giovanna Oliveira do Carmo, Érica Kaori Matsusaka Ijichi, Anna Giulia Correia Menino, Felipe Sanchez Bragagnolo, Mauricio Ariel Rostagno

**Affiliations:** Faculdade de Ciências Aplicadas (FCA), 28132 Universidade Estadual de Campinas (UNICAMP), Rua Pedro Zaccaria, 1300, Limeira, SP-CEP 13484-350, Brazil

## Abstract

The development of phenolic-rich functional ingredients, particularly those containing 5-caffeoylquinic acid (5-CQA), is limited by the use of conventional organic solvents, such as ethanol and methanol. Therefore, this study aimed to select and develop food-based solvents for the extraction of 5-CQA from green coffee and yerba mate, proposing a functional ingredient that maintains its composition after thermal processing for food and pharmaceutical applications. Mixtures of citric acid, sorbitol, and betaine, already widely used as ingredients with preservative, sweetening, and nutritional properties, were selected on the basis of COSMO-RS predictions for 5-CQA extraction. Different molar ratios of three solvent systems containing 10% water, betaine:sorbitol (1:1, 1:2, 1:3, 1:4, and 1:5), betaine:citric acid (1:1, 1:2, and 2:1), and the ternary system betaine:sorbitol:citric acid (1:1:1) were employed in the extractions. Among the evaluated systems, the 1:5 betaine:sorbitol, 1:2 betaine:citric acid, and ternary mixtures were effective for bioactive compound extraction and displayed DSC thermograms consistent with the formation of deep eutectic solvents (DESs). The 1:2 betaine:citric acid showed the highest extraction efficiency for 5-CQA, likely because of its lower pH (2.6). An acidic medium helps to retain its protons, preventing oxidation or isomerization. Phenolic compounds in coffee extracts were thermally stable in all solvents except 1:5 betaine:sorbitol, which may be due to its less acidic pH (5.95). Compounds in yerba mate extracts produced with 85:15 water:ethanol showed isomerization of 3-CQA into 5-CQA, whereas the formulated solvents reduced the thermal effect. Thus, food-based DESs efficiently extracted and thermally protected 5-CQA, showing strong potential for functional food applications.

## Graphical abstract

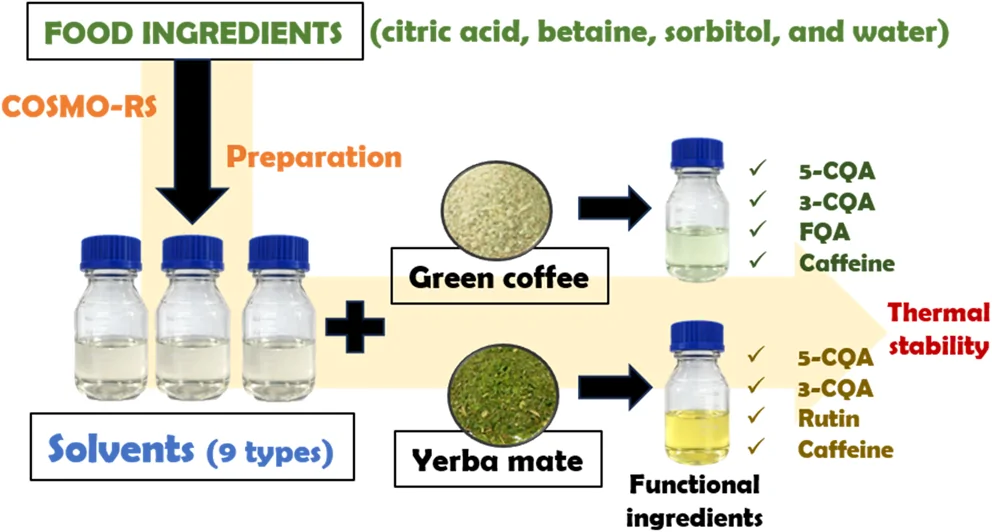

## 1 Introduction

Chlorogenic acid, also known as 5-caffeoylquinic acid (5-CQA), is one of the most abundant phenolic compounds in plants; coffee is its primary dietary source.^1^ Intake of 5-CQA has been negatively correlated with the risk of several adverse conditions, including oxidative and inflammatory stress, metabolic syndrome, hepatic steatosis, acute lung injury, neurodegenerative and cardiovascular diseases, gastrointestinal dysfunctions, and cancer.^2^ Only a fraction of ingested 5-CQA reaches systemic circulation as it undergoes extensive metabolism in the gastrointestinal tract and liver. Given its broad health effects, developing products enriched with this phenolic compound is a promising strategy to enhance its benefits.^3^

Numerous studies have investigated natural sources of 5-CQA, highlighting Arabica green coffee and yerba mate (*Ilex paraguariensis*), which contain high levels of this compound and are widely consumed as infusions.^4–6^ Coffee, one of the most important global commodities, is cultivated by more than 25 million farmers across 60+ countries, with Brazil as the world’s largest producer.^7^ Yerba mate, in turn, is native to the subtropical and temperate regions of South America and is predominantly cultivated in Argentina, Paraguay, and Brazil. In addition to 5-CQA, green coffee and yerba mate contain a variety of aromatic compounds highly appreciated by consumers. These constituents not only confer health-promoting properties but also enhance the sensory attributes of the food products in which they are incorporated, adding value and expanding their potential for application in the food industry.

Hydroethanolic mixtures have demonstrated superior performance for the extraction of phenolic compounds, and specifically chlorogenic acid from coffee materials.^8^ However, the use of these solvents introduces an additional separation step, as solvent removal is required before extract application. Therefore, the development of “ready-to-use” solvents with high selectivity and efficiency for the target compound, composed of substances that can be directly incorporated as ingredients in food and pharmaceutical products, is highly desirable.^9^

The use of in silico tools for solvent development and selection is crucial to this process. The conductor-like screening model for real solvents (COSMO-RS) is a computational method for predicting the thermophysical properties of mixtures based on quantum-mechanical calculations. It has been widely applied to describe the solubility of specific compounds in solvents and to assist in the design of deep eutectic solvents (DESs), suggesting mixture compositions with enhanced solubilization potential for target molecules.^10,11^ However, the software cannot reliably predict whether a combination of specific compounds will form DES. Therefore, it is necessary to formulate and characterize the solvent systems experimentally to confirm the formation of DESs. DESs are mixtures usually formed through the complexation of a hydrogen bond acceptor (HBA) and a hydrogen bond donor (HBD), exhibiting a melting point significantly lower than those of their individual constituents due to strong deviations from ideality.^12^ These interactions create a structured network that promotes the solvation of specific classes of compounds through multiple intermolecular interactions. Beyond their high solvation capacity and extraction efficiency, a key advantage of DESs is their potential to be composed of naturally derived, food-grade constituents, enabling direct incorporation into food products without solvent removal.

In addition to acting as extraction solvents, DES can also protect bioactive compounds by serving as stabilizer agents.^13,14^ The proposed form of protection is based on the HBD interaction with HBA, which can act as a substitute in anion or cation exchange when the compounds are exposed to oxidation conditions, such as light, heat, or storage time.^15^ Compared with conventional solvents, the main advantage of food-based DESs is that solvent evaporation is not required, reducing downstream processing steps. However, DESs are generally more expensive than hydroethanolic mixtures or water, and therefore, their composition should be selected to provide additional functional properties to the extract. In this context, citric acid, widely used in food, pharmaceutical, cosmetic, and chemical formulations as a preservative due to its chelating and sequestering properties, can confer this activity to the extracts.^16^ Sorbitol, commonly used as a sweetener, humectant, sequestrant, texturizer, stabilizer, and bulking agent, may improve the sensory properties of bioactive-rich extracts.^17^ Furthermore, betaine contributes to nutritional and functional value through its bioactive properties, including cellular hydration, methylation balance, antioxidant defense, and metabolic regulation.^18^

Accordingly, this study aimed to select and develop food-based solvents using citric acid, betaine, and sorbitol for the extraction of 5-CQA from green coffee and yerba mate, proposing a functional ingredient that maintains its composition after thermal processing for food and pharmaceutical applications.

## 2 Material and Methods

### 2.1 Materials

Arabica green coffee and yerba mate (*Ilex paraguariensis*) were purchased from a local store in Limeira, São Paulo. They were ground, and the fractions retained on Tyler sieves with diameters of 180, 300, and 500 mm were mixed and used as raw materials in this study. Solvents were produced with betaine (Sigma-Aldrich, ≥ 98% purity), sorbitol (Dinâmica, 94% purity), citric acid (Sigma-Aldrich, 100% purity), xylitol (Alamar Tecno-Científica Ltd.a, 99% purity), methionine (Alamar Tecno-Científica Ltd.a, 99% purity), tryptophan (Alamar Tecno-Científica Ltd.a, 99.7% purity), and (±)-α-tocopherol (Sigma-Aldrich, ≥ 96% purity).

### 2.2 Solvent Selection by COSMO-RS and Preparation

The COSMO-RS method was used to select food-based solvents for the extraction of 5-CQA from green coffee and yerba mate. The components used in COSMO-RS predictions were selected from compounds with potential applications in food, enabling the production of ready-to-use extracts without solvent removal. These compounds were selected from the databases presented by Omar and Sadeghi^19^ and Bragagnolo et al.,^9^ which compile potential natural compounds for the formulation of DES. Thus, the compounds selected were: choline chloride, sorbitol, ribitol, xylitol, fructose, glucose, sucrose, maltose, dextrose, lysine, methionine, cysteine, tryptophan, betaine, l-threonine, lactic acid, citric acid, malic acid, inulin, ergocalciferol, cobalamin, riboflavin, and α-tocopherol. Geometry optimization and molecular charge density for the COSMO-RS assessments were performed using the Turbomole software (TmoleX 2022 Version 22.0.0) under the COSMO-BP-TZVP specification. These calculations were conducted using the COSMOtherm package (Version 22.0.0) with the BP_TZVP_22 settings.

For solvent screening, more than 1000 component combinations were generated by pairing the natural constituents. Combinations of each compound were chosen based on the infinite dilution activity coefficient values (ln γ^∞^), as the potential to solubilize 5-CQA. The solvents were prepared by weighing component mass to acquire a molar ratio of 1:1 (molar ratio) in a capped reagent bottle and heated from 60 to 100 °C under magnetic stirring until no insoluble compounds were observed. Solvents were stored at room temperature (25 ± 2 °C) for 24 h to verify their stability.

### 2.3 Solvents Characterization

#### 2.3.1 Viscosity

The viscosity of solvents was measured using a Brookfield LVDV1 viscometer (Brookfield Engineering Laboratories, USA) equipped with an SC4–34 cone spindle and a UL adapter. Measurements were performed at a constant rotational speed of 0.5 rpm under oscillatory shear. Viscosity values were recorded in centipoise (cP).

#### 2.3.2 Differential Scanning Calorimetry

Differential scanning calorimetry (DSC) analyses of the solvents and their individual constituents were performed using a DSC1 (Mettler Toledo, Schwerzenbach, Switzerland). Approximately 6–8 mg of each pure component (betaine, citric acid, and sorbitol) was weighed into aluminum pans sealed with lids and analyzed over a temperature range from −40 to 300 °C at a heating rate of 10 °C/min, after equilibration for 3 min at – 40 °C under a nitrogen atmosphere (50 mL/min), according to Benvenutti et al.^20^ Using this protocol, the melting points of the mixtures could not be detected; therefore, an alternative method was applied. The samples were cooled from 25 to −100 °C at 2 °C/min, held at −100 °C for 10 min, and subsequently heated to 140 °C at 10 °C/min under a nitrogen atmosphere (50 mL/min).^20^

### 2.4 Solid–Liquid Extractions

Solid–liquid extractions were performed using a C-MAG HS IKA magnetic hot-plate stirrer, with simultaneous heating and magnetic stirring, under fixed conditions to observe the effect of the solvent on the extraction. Briefly, 1.5 g of ground green coffee or yerba mate was added to 15 g of solvent, with a solvent-feed (S/F) ratio of 10 (w/w), using a magnetic stirrer in a 50 mL Becker. The selected S/F was determined based on preliminary experimental conditions that ensured adequate homogenization and handling of the extraction systems, particularly given the relatively high viscosity of solvents. The extraction system was heated under continuous stirring at 300 rpm until the internal extraction temperature reached 70 °C. Once this temperature was reached, the 5 min extraction time began. The binomial time–temperature was chosen to reduce solvent viscosity while avoiding thermal degradation of the extracted compounds. Based on previous studies conducted by our research group, as well as reports available in the literature, relatively high temperatures (∼70 °C) combined with short extraction times (up to 5 min) allow efficient extraction of 5-CQA while minimizing the thermal degradation observed under more severe conditions, particularly at temperatures closer to 100 °C and longer heating periods.^21–23^

After extraction, 10 g of distilled water was added to the mixture, followed by manual homogenization with a spatula to reduce the viscosity and facilitate filtration. The extracts were then filtered by using filter paper to remove the solid residue. All experiments were performed in triplicate for each of the solvent systems.

### 2.5 Thermal Stability of the Extracts Obtained in Different Solvents

The thermal stability test was performed at 120 °C, as described by Strieder et al.^24^ The extracts were lyophilized and subsequently solubilized in distilled water at a concentration of 10 mg/mL. The solutions were analyzed according to Section 2.6 before and after heating at 120 °C in an oven for 1 h. However, the stability results were reported as μg of extracted compound/mL of solution. The pH of the solutions was also measured.

### 2.6 Identification and Quantification of Bioactive Compounds

The quantification of compounds extracted from green coffee was performed by ultrahigh performance liquid chromatography (UHPLC) coupled to a photodiode array detector (PDA), according to Strieder et al.^25^ For yerba mate samples, the method described by Souza et al.^26^ was employed. Compound separation was performed in a Kinetex C18 column (100 × 4.6 mm i.d., 2.6 μm; Phenomenex, Torrance, CA, USA) at a flow rate of 1.0 mL/min. The mobile phase consisted of 0.1% (v/v) acetic acid in water (solvent A) and 0.1% (v/v) acetic acid in acetonitrile (solvent B). Detection and quantification were performed at 270 and 350 nm. Chromatograms were recorded at both wavelengths, and calibration curves were prepared using 5-caffeoylquinic acid (7.75–300 ppm) and caffeine (12.6–100 ppm) for quantification of the compounds. Extraction yields were reported as mg of extracted compound/g of raw material used in the extraction (green coffee or yerba mate).

Identification of additional compounds was tentatively performed using a liquid chromatography–mass spectrometry (LC–MS) system comprising an ESI–IT–TOF mass spectrometer (Shimadzu, Japan) coupled to a UFLC system (20A Prominence, Shimadzu). Extracts were injected into a Kinetex C18 column (5 μm; 100 × 2.1 mm) and eluted using optimized binary solvent systems for each ionization mode. For positive ionization mode, the solvents consisted of (A) water:acid (999:1, v/v) and (B) acetonitrile:water:acid (900:99:1, v/v/v). For negative ionization mode, the acid was omitted to enhance ionization efficiency, and the solvents used were (A) water and (B) acetonitrile:water (900:100, v/v). A linear gradient from 0 to 100% solvent B was applied over 20 min at a constant flow rate of 0.25 mL/min. Samples were initially monitored using a photodiode array detector (SPD-M20A, Shimadzu) before introduction into the mass spectrometer. Mass spectrometry parameters were as follows: interface voltage, 4.5 kV; capillary voltage, 1.95 kV; temperature, 200 °C; and collision-induced dissociation with argon at 70% energy. MS spectra were acquired in the *m*/*z* range of 200–1500, and MS/MS spectra in the range of 100–1400 *m*/*z*. Analyses were performed sequentially in positive and negative ionization modes to ensure comprehensive detection of molecular components present in the samples.

### 2.7 Statistical Analysis

The mean difference was verified using analysis of variance (ANOVA) in Minitab 18 with a 95% confidence level (*p*-value ≤ 0.05). Tukey’s test of means was performed at a 95% confidence level (*p*-value ≤ 0.05).

## 3 Results and Discussion

### 3.1 Compounds Identification

Figure S1 of the Supporting Information presents the chromatograms of the extracted compounds from green coffee and yerba mate. The 5-CQA and caffeine were identified in the extracts by analysis of the compound standards. The 3-caffeoylquinic acid (3-CQA) and quinic acid derivatives (FQA) in coffee extracts (Figure S1A) were tentatively identified by the ESI-IT-TOF, presenting the negative mass charges of 353.08 *m*/*z* (with MS/MS 191.05 and 179.03) and 367.10, respectively. The negatively charged mass of 353.08 with MS/MS of 191.05 and 179.03 is characteristic of 3-CQA, while the 367 *m*/*z* is characteristic of quinic acid derivatives (3-O-feruloyl-D-quinic acid or 4-O-feruloyl-D-quinic acid) as identified in previous studies.^27,28^ The peaks labeled as compounds C5, 6, and 7 were not identified because the ESI-IT-TOF peaks did not precisely match those observed in the UV–vis spectrum. However, one of the observed charge masses among these retention times, corresponding to compounds 5, 6, and 7, was 515.11, which may correspond to 3,4/3,5/4,5-di-O-caffeoylquinic acids (DCQA), compounds also typically found in coffee and its residues.^29^

In yerba mate extracts (Figure S1B), rutin and 3,5-dicaffeoylquinic acid (3,5-diCQA) were tentatively identified by ESI–IT–TOF, showing deprotonated ions at *m*/*z* 609.14 and 515.11, respectively. The 4,5-dicaffeoylquinic acid (4,5-diCQA) was tentatively identified based on its UV–Vis spectrum, which showed characteristic absorption maxima (λmax) at 216.8, 243.7, and 326.9 nm. These compounds have been previously reported in yerba mate extracts.^26^

### 3.2 Solvent Selection, Formation, and Viscosity

The solvent compositions proposed by the COSMO-RS and their infinite dilution activity coefficients (ln γ∞) concerning 5-CQA, were as follows: betaine:sorbitol 1:1 (−21.68), methionine:xylitol 1:1 (−20.60), betaine:methionine:sorbitol 0.33:0.33:0.33 (−20.43), betaine:methionine 1:1 (−20.08), tocopherol:betaine:sorbitol 0.33:0.33:0.33 (−17.35), citric acid:betaine 1:1 (−15.44), citric acid:betaine:sorbitol 0.33:0.33:0.33 (−15.33), citric acid:betaine:methionine 0.33:0.33:0.33 (−14.97), and betaine:tryptophan:sorbitol 0.33:0.33:0.33 (−14.88). However, experimentally, none formed a liquid phase. Since the objective of this study was to develop liquid solvent systems for extraction applications, 10% (w/w) water was incorporated to evaluate its ability to promote the formation and stabilization of a homogeneous liquid phase suitable for the extraction process. After this adjustment, three mixtures remained in the liquid state after 24 h of preparation: betaine:sorbitol (1:1), citric acid:betaine (1:1), and citric acid:betaine:sorbitol (1:1:1), all containing 10% added water.

In addition to these mixtures, the solvents were formulated at other molar ratios. For betaine:sorbitol, ratios of 1:1, 1:2, 1:3, 1:4, and 1:5 were prepared, while for citric acid:betaine, ratios of 1:1, 1:2, and 2:1 were formulated, all containing 10% added water. The betaine:sorbitol mixtures at molar ratios of 1:1, 1:2, 1:3, 1:4, and 1:5 exhibited viscosities of 17,405 ± 333, 19,800 ± 509, 19,380 ± 933, 42,780 ± 594, and 72,960 ± 679 cP, respectively, measured at 20 ± 2 °C. These results indicate a clear increase in viscosity with increasing sorbitol content in the betaine:sorbitol formulations. In contrast, betaine:citric acid mixtures at molar ratios of 1:1, 1:2, and 2:1 exhibited viscosities higher than 120,000, 103,800, and 94,929 cP, respectively, measured at 33 ± 3 °C. Additionally, the ternary citric acid:betaine:sorbitol system (1:1:1) exhibited a viscosity of 78,300 ± 1,103 cP at 20 ± 2 °C. These viscosities were significantly higher than those observed for conventional ethanol–water mixtures, whose viscosity at 25 °C is on the order of 0.89 cP for water, around 1.1 cP for ethanol, and up to approximately 2.3 cP for hydroalcoholic mixtures, depending on the proportion between the components.^30^

### 3.3 Solvent Effects on the Extraction of Bioactive Compounds

Figures 1 and 2 present the performance of the developed solvents for the extraction of compounds from green coffee and yerba mate, respectively. Overall, a similar extraction trend was observed for 3-CQA, 5-CQA, FQA, and caffeine across all solvent systems, likely due to their comparable, relatively high polarity. Among the evaluated systems, the highest extraction efficiencies were obtained with solvents formulated with betaine and citric acid. These were followed by betaine–sorbitol mixtures and the ternary system (betaine:citric acid:sorbitol), which showed only minor differences among them. Notably, the control solvent (ethanol:water, 15:85 w/w) exhibited extraction performance comparable to that of the betaine–sorbitol mixtures.

**Figure 1:**
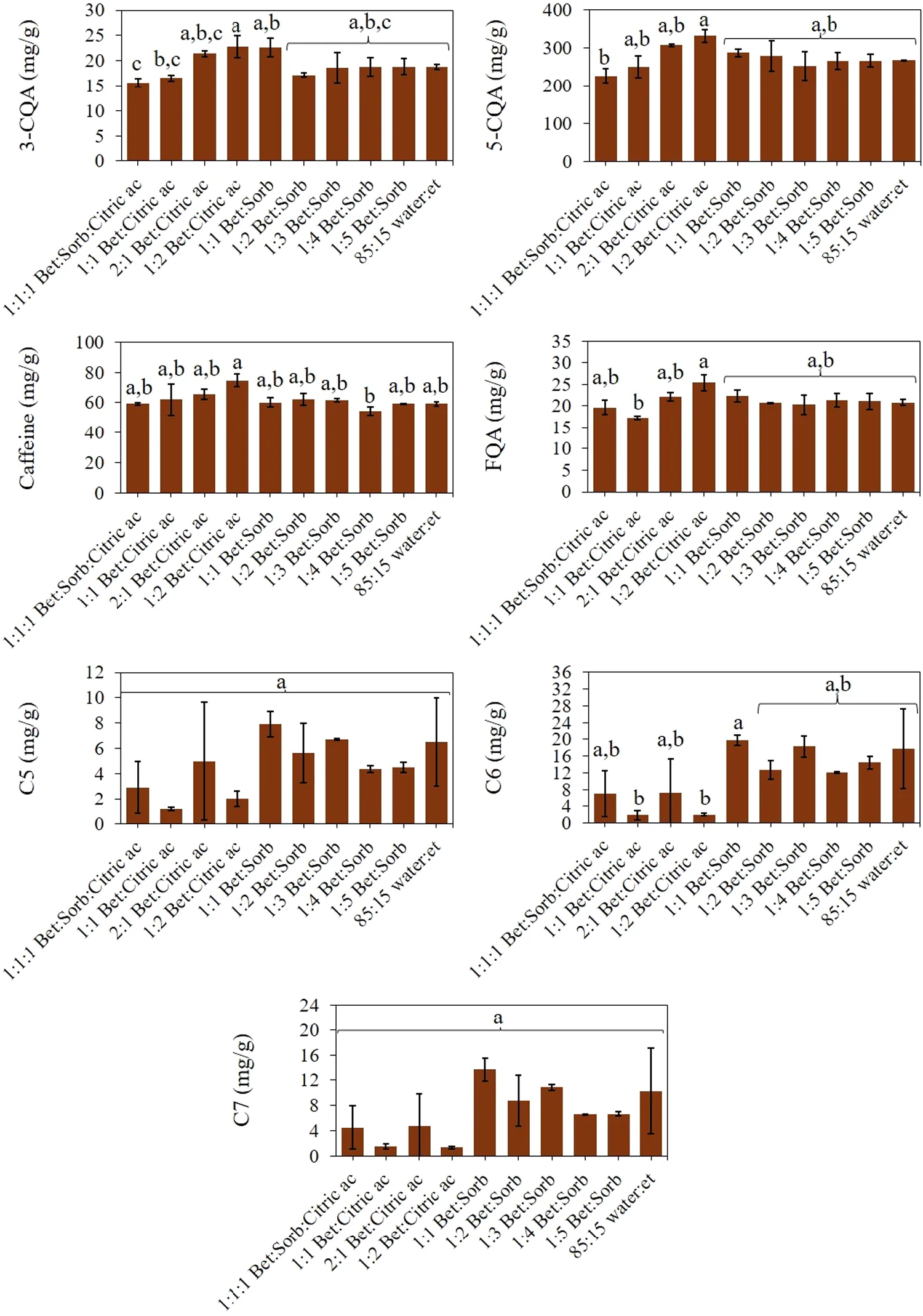
Effect of the solvent on the extraction of 5-CQA, 3-CQA, caffeine, FQA, and other unidentified compounds (C5, C6, and C7) from green coffee. Bars labeled with different letters indicate statistically significant differences according to Tukey’s test at a 95% confidence level.

**Figure 2:**
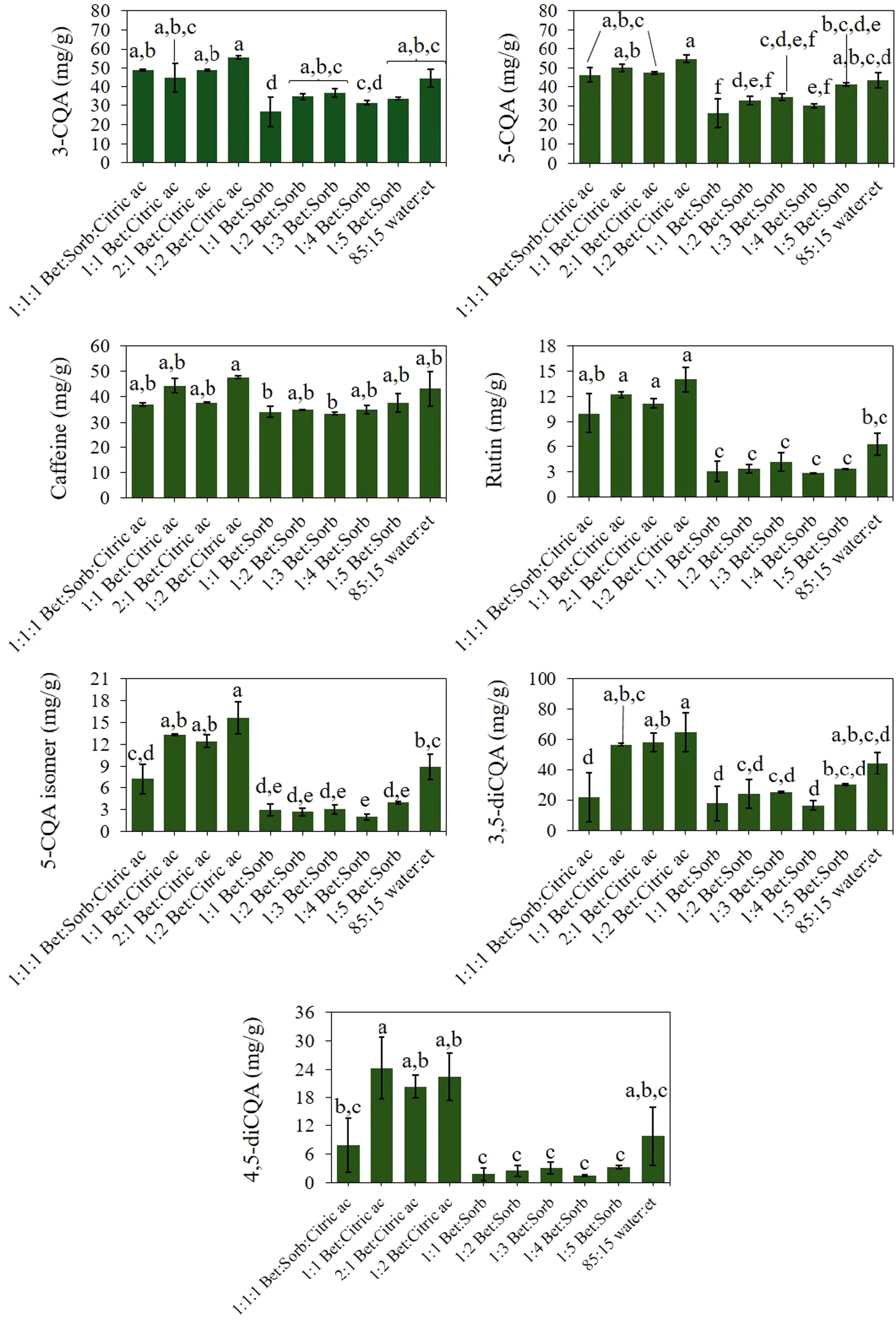
Effect of the solvent on the extraction of 3-CQA, 5-CQA, caffeine, rutin, 5-CQA isomer, 3,5-diCQA, and 4,5-diCQA from yerba mate. Bars labeled with different letters indicate statistically significant differences according to Tukey’s test at a 95% confidence level.

Although lower-viscosity solvents are generally expected to enhance mass transfer by facilitating more solvent–solute interactions, this trend was not observed in the present study as the hydroethanolic mixture did not perform better than the developed high-viscosity solvents. Furthermore, solvents based on betaine and citric acid exhibited the highest viscosities (94,929 to >120,000 cP at 33 ± 3 °C) and the best extraction performance. Similarly, the 1:5 molar ratio of betaine:sorbitol presented the highest viscosity (72,960 ± 679 cP) and the best extraction efficiency for yerba mate compounds. These findings indicate that viscosity was not the determining factor governing extraction efficiency. However, it should be noted that viscosity measurements were performed at approximately 20 °C, whereas the extraction experiments were conducted at 70 °C. Therefore, the higher extraction temperature likely promoted a substantial reduction in solvent viscosity, as reported by Gajardo-Parra^31^ for a similar betaine:sorbitol:water (1:1:3) system.

Therefore, the superior extraction performance was more closely associated with their specific solvent–solute interactions and physicochemical affinity for the target compounds than with viscosity alone. Solvent properties such as polarity and solvation capacity may play a more decisive role. COSMO-RS predictions further support this theory. According to the computational analysis, the betaine:sorbitol (1:1) system was predicted to exhibit the highest affinity for 5-CQA, as indicated by ln γ∞ values. However, experimentally, the highest extraction efficiency was achieved with the betaine:citric acid (1:2) system. This discrepancy can be explained by the σ-profile and σ-potential distributions (Figure 3), which show that citric acid presents a polarity

**Figure 3:**
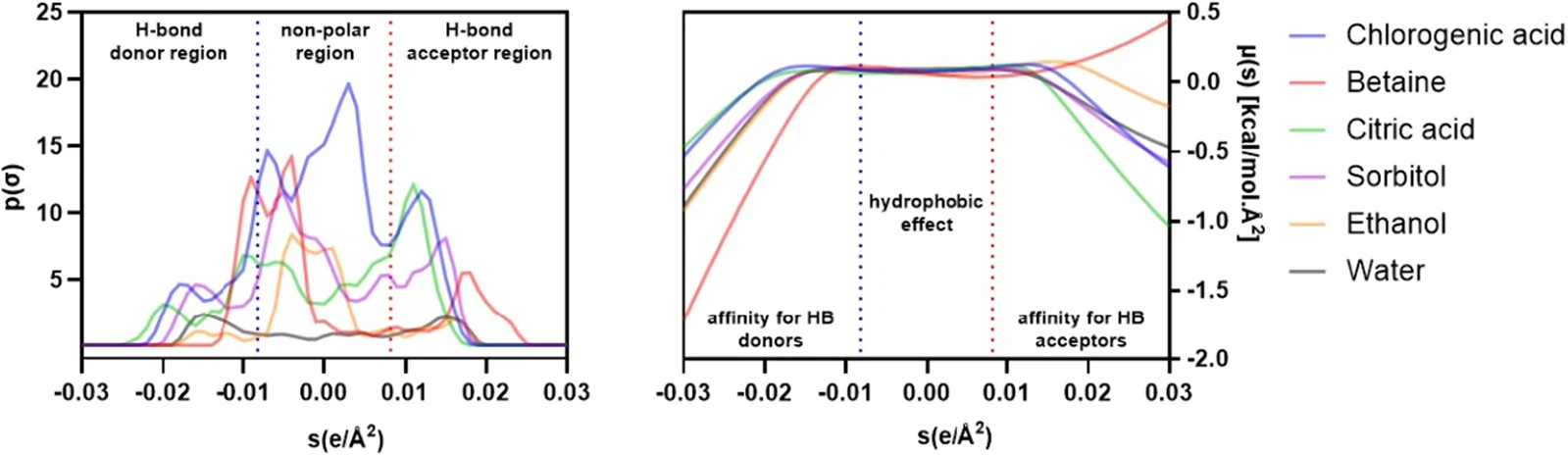
Profile and potential σ generated by COSMO-RS for 5-CQA and solvent components.

profile more similar to that of 5-CQA, favoring stronger solvent–solute interactions and enhanced solubilization. Additionally, the more acidic environment provided by this system likely contributed to the increased stability of chlorogenic acids (Table 1).^32^

**Table 1:** Solvent Characterization Regarding pH and Melting Point

solvent	pH	melting point (DSC)
1:5 betaine:sorbitol	4.63 ± 0.01	–34.76 °C
1:2 betaine:citric acid	1.80 ± 0.02	–60.65 °C
1:1:1 betaine:sorbitol:citric acid	2.67 ± 0.01	–40.93 °C

This dependence on solvent polarity and solubility also influenced the extraction selectivity. Solvents without citric acid, or with lower citric acid content (as in the ternary system), were more effective for extracting rutin, a 5-CQA isomer, 3,4-diCQA, and 4,5-diCQA from yerba mate. In contrast, solvents with a higher citric acid content were more efficient at extracting C5, C6, and C7 from green coffee.

These findings are consistent with previous studies. Ruesgas-Ramón et al.^33^ and Yue et al.^34^ also reported higher extraction yields of 5-CQA using acidic DES systems, such as choline chloride:lactic acid (2:1) and proline:malic acid (1:1), applied to coffee coproducts and *Artemisia scoparia*, respectively. In those studies, the improved extraction efficiency was attributed to strong intermolecular interactions between the DES and 5-CQA, as well as to the stabilizing effect of acidic media, which can help prevent compound degradation. Furthermore, the observed extraction yields were within the expected range for these plant matrices and, in some cases, exceeded previously reported values. Rojas-González et al.^35^ reported total yields ranging from 41 to 113 mg/g for the sum of CQAs, FQAs, and DCQAs extracted from green coffee, and 91.9 mg/g for the sum of 3-CQA, 4-CQA, 5-CQA, 3,4-DQA, 3,5-DQA, and 4,5-DQA extracted from yerba mate. In the present study, the sum of phenolic compounds extracted from green coffee ranged from 276 mg/g using betaine:sorbitol:citric acid (1:1:1) to 386 mg/g using betaine:citric acid (1:2). For yerba mate, the sum ranged from 75 mg/g using betaine:sorbitol (1:1) to 189 mg/g using betaine:citric acid (1:1), yielding values also higher than those observed by Souza et al.^26^ of around 70 mg/g using water and ethanol as solvents.

Raw material characteristics, other than particle size, such as composition, moisture content, and morphology, may also influence solvent performance and extraction efficiency.^23^ However, in the present study, a similar extraction behavior was observed for the same solvent systems when extracting the same target compounds (3-CQA, 5-CQA, and caffeine) from different plant matrices (green coffee and yerba mate). In this case, the solvent affinity and solubility toward specific compounds appeared to exert a more pronounced effect on the extraction efficiency than the intrinsic characteristics of the matrices, which could influence the interactions between the target compounds and the plant structure. Therefore, given that the extraction yields obtained for both matrices were generally higher than those reported in the literature, the developed solvent systems demonstrated considerable potential and, in several cases, outperformed conventional extraction solvents. Moreover, their effectiveness with both green coffee and yerba mate indicates their suitability for extracting similar chlorogenic acid derivatives from different plant matrixes.

### 3.4 Selected Solvents Characterization and Thermal Stability

To compare the three types of formulated solvents in terms of the melting point and thermal protection properties, one representative formulation from each system was selected. From the betaine:citric acid mixtures, the 1:2 ratio was selected for its superior performance. The 1:5 betaine:sorbitol formulation was selected for its higher extraction efficiency for certain compounds and lower cost due to the higher proportion of sorbitol relative to betaine. Finally, the 1:1:1 betaine:sorbitol:citric acid ternary mixture was included for comparison. Table 1 presents the characterization of the three solvents, and Figure 4 presents the DSC thermograms.

**Figure 4:**
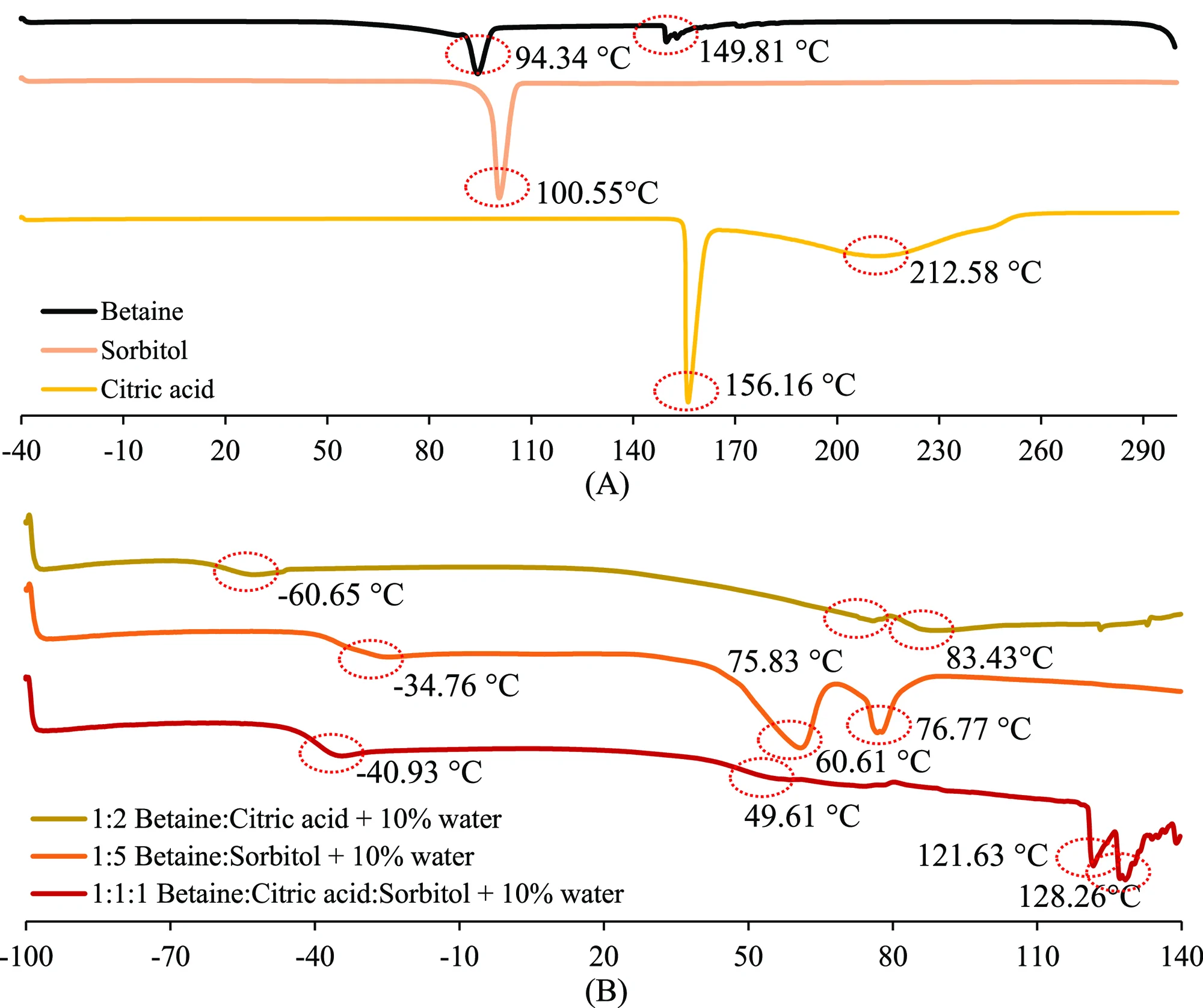
DSC thermograms obtained for the solvents (B) and their individual components (A).

The DSC thermograms presented in Figure 4 indicate the potential formation of DES in all three formulated mixtures, as evidenced by the pronounced reduction in melting temperature compared to their pure components. This behavior is characteristic of strong intermolecular interactions, particularly hydrogen bonding, which are typical of DES systems. Additional thermal events were observed in the mixtures at temperatures >49.61 °C, which may be associated with structural relaxations, molecular rearrangements, and water loss. Thermal events above 100 °C may be associated with the onset of thermal decomposition or structural instability following molecular reorganization.

Figures 5 and 6 present, respectively, the thermal stability of green coffee and yerba mate extracts in the different solvents after thermal treatment at 120 °C for 1 h, and Table 2 presents the pH values of each extract. The difference in compound content observed between the thermal stability test (Figures 5 and 6) and the extraction yield results (Figures 1 and 2) is due to differences in sample preparation. For the thermal stability assay, all samples were freeze-dried, and the same mass was weighed for analysis. Thus, in the extracts obtained with the conventional solvent (85:15 water:ethanol), complete solvent evaporation after freeze-drying left only the solids extracted from coffee or yerba mate. In contrast, extracts obtained using DES retained both the DES constituents and the extracted compounds. Consequently, when redissolved, these samples exhibited lower apparent concentrations of the target compounds due to dilution by the DES matrix.

**Figure 5:**
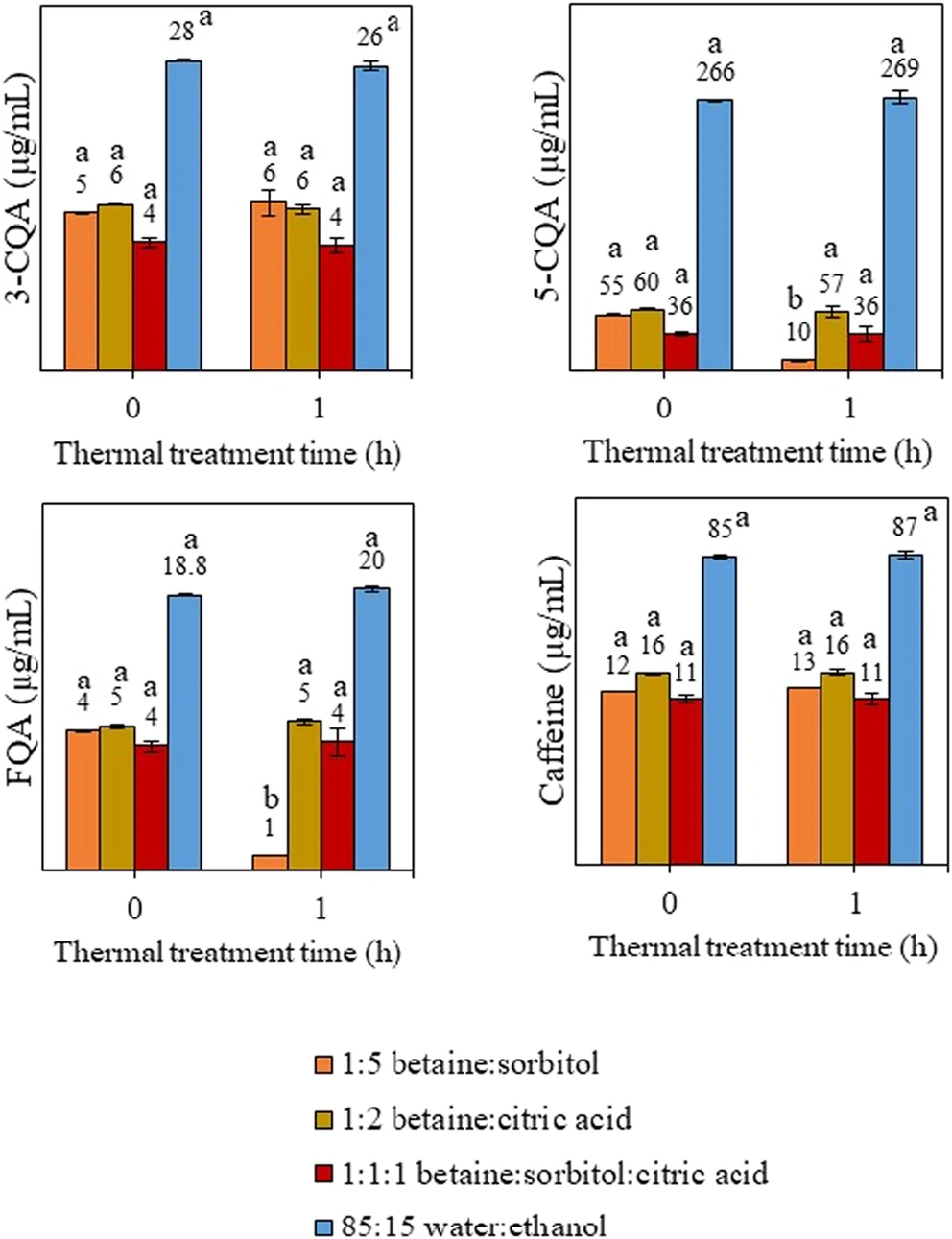
Thermal stability of green coffee extracts in different solvents after thermal treatment at 120 °C for 1 h.

**Figure 6:**
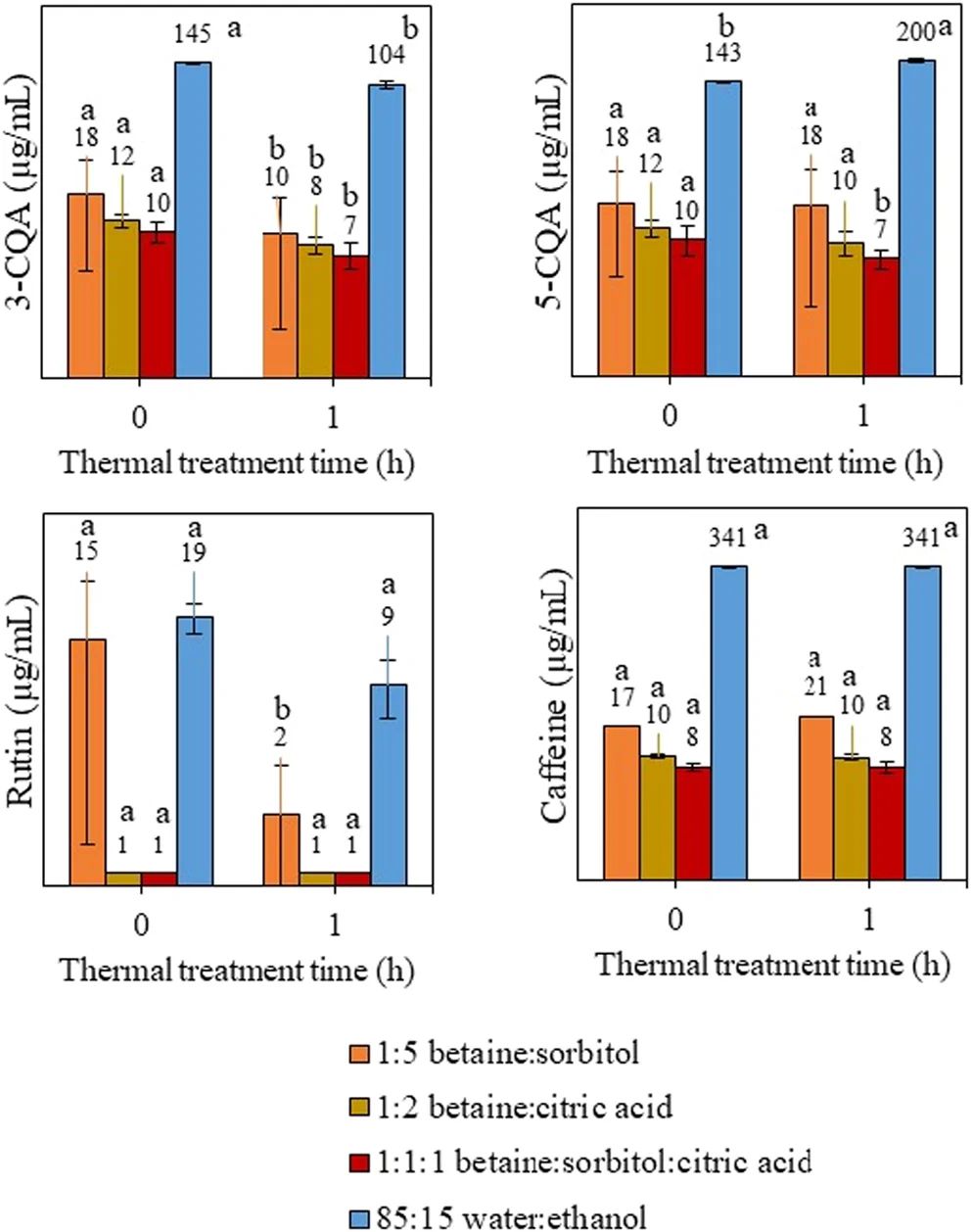
Thermal stability of yerba-mate extracts in different solvents after thermal treatment at 120 °C for 1 h.

**Table 2:** pH Value of Extracts

solvent	green coffee extract pH	Yerba mate extract pH
1:5 betaine:sorbitol	5.59 ± 0.01	5.95 ± 0.04
1:2 betaine:citric acid	2.61 ± 0.01	2.60 ± 0.02
1:1:1 betaine:sorbitol:citric acid	2.81 ± 0.01	2.73 ± 0.01
85:15 water:ethanol	3.00 ± 0.01	5.63 ± 0.03

The coffee compounds exhibited high stability under applied thermal treatment. In the coffee extracts, except for the 1:5 betaine:sorbitol formulation, all samples remained stable after heating (Figure 5). This behavior is likely associated with the pH of the extracts (Table 2) as chlorogenic acids are more stable at lower pH values. Under acidic conditions, the phenolic hydroxyl groups remain protonated, making them less susceptible to oxidation, hydrolysis, and isomerization.

On the contrary, the bioactive compounds in yerba mate were more susceptible to thermal degradation. The 3-CQA content decreased to approximately 70% (m/m) of its initial value in all solvents, except in the 1:5 betaine:sorbitol system, where it was further reduced to about 40% (m/m). The 3-CQA likely underwent isomerization to 5-CQA in the extract obtained with the 85:15 water:ethanol solvent, as the 5-CQA content after thermal treatment was 140 times higher than that before treatment. In the DES systems, the 5-CQA content decreased to approximately 70% (w/w) of its initial value. However, a significant difference was observed only in the extract obtained with the 1:1:1 betaine:sorbitol:citric acid system. The rutin content decreased in the extracts obtained with the 85:15 water:ethanol mixture and was completely degraded in the 1:5 betaine:sorbitol system, whereas it was preserved in DES containing citric acid.

Caffeine was the only compound whose content remained unchanged after thermal treatment, demonstrating its thermal stability regardless of the solvent used. Therefore, in addition to the three-dimensional structures formed by the DES, the pH of the mixtures containing citric acid favored the preservation of the compounds diluted in water after heat treatment at 120 °C.

## 4 Conclusion

Solvents based on food ingredients were successfully developed and demonstrated their efficiency in the extraction of phenolic compounds. They yielded bioactive compound recoveries comparable to or higher than those of the hydroethanolic mixture (85:15 water:ethanol) from green coffee and yerba mate. Among the evaluated systems, the 1:5 betaine:sorbitol, 1:2 betaine:citric acid, and the ternary mixture exhibited DSC thermograms consistent with DES formation, as evidenced by significant melting point depression. The 1:2 betaine:citric acid system showed the highest extraction efficiency for 5-CQA, likely because of its lower pH (2.6). Furthermore, phenolic compounds in coffee extracts were thermally stable in all solvents except 1:5 betaine:sorbitol, possibly due to its higher (less acidic) pH (5.95). In contrast, compounds in yerba mate extracts were more sensitive to thermal treatment, particularly in the conventional solvent (85:15 water:ethanol), where the isomerization of 3-CQA into 5-CQA was observed. In this context, the formulated DESs based on citric acid and betaine appeared to exert a protective effect against thermal degradation of yerba mate extracts. Overall, this study demonstrates the potential of food-grade DES as an efficient extraction medium for 5-CQA while also enhancing the thermal stability of bioactive compounds, supporting their application in functional food development.

## Supplementary Material



## Notes

Chromatograms of the extracted compounds from green coffee and yerba mate (PDF)

## References

[1] da
Silva W. B., Rocha L. M., Moura L. D. G., Soares M. S., da Silva S. A., Moreira D. B., Good God P. I. V., Silva G. H. (2025). Optimization
of methodology for simultaneous quantification of trigonelline, 5-caffeoylquinic
acid, and caffeine in green and roasted coffee extracts by HPLC. ACS Omega.

[2] Lu H., Tian Z., Cui Y., Liu Z., Ma X. (2020). Chlorogenic
acid: A comprehensive review of the dietary sources, processing effects,
bioavailability, beneficial properties, mechanisms of action, and
future directions. Compr. Rev. Food Sci. Food
Saf..

[3] Farah A., Monteiro M., Donangelo C. M., Lafay S. (2008). Chlorogenic acids from
green coffee extract are highly bioavailable in humans. J. Nutr..

[4] Bosso H., Maria B. S., Ricardo Otoboni d. A.
G., A M M. B., Otoboni (2023). Green coffee: economic
relevance and a systematic review of the effects on human health. Crit. Rev. Food Sci. Nutrit..

[5] Meinhart A. D., Da Silveira T. F. F., Godoy H. T. (2024). Yerba mate as an
inexpensive source
of analytical standards of chlorogenic acid isomers: an optimization
study. Food Analytical Methods.

[6] Panzl M. V., Menchaca D., Rodríguez-Haralambides A. (2022). Analysis of
polyphenols and xanthines in yerba mate (Ilex paraguariensis) infusions
by high-pressure extraction and ultra-high performance liquid chromatography. Applied Food Res..

[7] Pham Y., Reardon-Smith K., Mushtaq S., Cockfield G. (2019). The impact
of climate change and variability on coffee production: a systematic
review. Climatic Change.

[8] de
Aquino Cardoso Andrade M., Martins Strieder M., Lacerda Sanches V., Sanchez Bragagnolo F., Ariel Rostagno M. (2026). Enhancing
the extraction of bioactive compounds from coffee waste using thermosonication
combined with alternative solvents for ready-to-use products. ACS Food Sci. Technol..

[9] Bragagnolo F. S., Strieder M. M., Pizani R. S., de Souza Mesquita L. M., González-Miquel M., Rostagno M. A. (2024). Revisiting
natural
deep eutectic solvents (NADES) as extraction media and ready-to-use
purposes. TrAC, Trends Anal. Chem..

[10] Strieder M. M., Bragagnolo F. S., Mendiola J. A., Rostagno M. A., Ibáñez E. (2024). Screening
and characterization of 1,8-cineole-based solvents as an alternative
to hexane for obtaining nonpolar compounds from plant-based milk coproducts. ACS Sustainable Chem. Eng..

[11] Wojeicchowski J. P., Ferreira A. M., Abranches D. O., Mafra M. R., Coutinho J. A. (2020). Using COSMO-RS
in the design of deep eutectic solvents for the extraction of antioxidants
from rosemary. ACS Sustainable Chem. Eng..

[12] Benvenutti L., Zielinski A. A. F., Ferreira S. R. S. (2019). Which is the best food emerging solvent:
IL, DES or NADES?. Trends Food Sci. Technol..

[13] Jeliński T., Przybyłek M., Cysewski P. (2019). Natural Deep Eutectic Solvents as
Agents for Improving Solubility, Stability and Delivery of Curcumin. Pharm. Res..

[14] Barbieri J. B., Goltz C., Cavalheiro F. B., Toci A. T., Igarashi-Mafra L., Mafra M. R. (2020). Deep eutectic solvents
applied in the extraction and
stabilization of rosemary (Rosmarinus officinalis L.) phenolic compounds. Ind. Crops Prod..

[15] Socas-Rodríguez B., Torres-Cornejo M. V., Álvarez-Rivera G., Mendiola J. A. (2021). Deep eutectic
solvents for the extraction of bioactive compounds from natural sources
and agricultural by-products. Appl. Sci..

[16] Ksiązek E. (2024). Citric acid:
properties, microbial production, and applications in industries. Molecules.

[17] Silva M. M., Reboredo F. H., Lidon F. C. (2023). Sweetener
food additives: a synoptical
overview on their chemical properties, applications in food products
and side effects. Emirates J. Food Agriculture.

[18] Buonaiuto G., Federiconi A., Vecchiato C. G., Benini E., Mordenti A. L. (2025). Betaine
dietary supplementation: healthy aspects in human and animal nutrition. Antioxidants.

[19] Omar K. A., Sadeghi R. (2023). Database of deep eutectic
solvents and their physical
properties: A review. J. Mol. Liq..

[20] Benvenutti L., Sanchez-Camargo A. d. P., Zielinski A. A. F., Ferreira S. R. S. (2020). NADES as potential
solvents for anthocyanin
and pectin extraction from Myrciaria cauliflora fruit by-product:
In silico and experimental approaches for solvent selection. J. Mol. Liq..

[21] De
Maria C. A. B., Trugo L. C., De Mariz e Miranda L. S., Salvador E. (1998). Stability of 5-caffeoylquinic acid under different
conditions of heating. Food Res. Int..

[22] Mangiapelo L., Blasi F., Ianni F., Barola C., Galarini R., Abualzulof G. W., Sardella R., Volpi C., Cossignani L. (2023). Optimization
of ultrasound-assisted extraction of chlorogenic acid from potato
sprout waste and enhancement of the in vitro total antioxidant capacity. Antiocidants.

[23] Strieder M. M., Velásquez Piñas J. A., Ampese L. C., Costa J. M., Carneiro T. F., Rostagno M. A. (2023). Coffee
biorefinery: The main trends
associated with recovering valuable compounds from solid coffee residues. J. Cleaner Prod..

[24] Strieder M. M., Bragagnolo F. S., Pizani R. S., Rostagno M. A. (2024). The impact
of using
an expansion gas with high-intensity ultrasound on the pressurized
liquid extraction of bioactive compounds from an industrial coffee
solid residue. Innovative Food Sci. Emerging
Technol..

[25] Strieder M. M., Sanches V. L., Rostagno M. A. (2024). Simultaneous extraction, separation,
and analysis of 5-caffeoylquinic acid and caffeine from coffee co-product
by PLE-SPE × HPLC-PDA two-dimensional system. Food Res. Int..

[26] Souza M. C., Silva L. C., Chaves J. O., Salvador M. P., Sanches V. L., da Cunha D. T., Carneiro T. F., Rostagno M. A. (2021). Simultaneous
extraction
and separation of compounds from mate (Ilex paraguariensis) leaves
by pressurized liquid extraction coupled with solid-phase extraction
and in-line UV detection. Food Chem.: Molecular
Sci..

[27] Willems J. L., Khamis M. M., Mohammed
Saeid W., Purves R. W., Katselis G., Low N. H., El-Aneed A. (2016). Analysis of a series of chlorogenic
acid isomers using differential ion mobility and tandem mass spectrometry. Anal. Chim. Acta.

[28] Machado M., Espírito Santo L., Machado S., Lobo J. C., Costa A. S., Oliveira M. B. P., Ferreira H., Alves R. C. (2023). Bioactive
potential and chemical composition of coffee by-products: From pulp
to silverskin. Foods.

[29] Clifford M. N., Wight J. (1976). The measurement of feruloylquinic acids and caffeoylquinic acids
in coffee beans. Development of the technique and its preliminary
application to green coffee beans. J. Sci. Food
Agric..

[30] Zhang, B. ; Liu, H. ; Dong, J. ; Liu, S. Effect of temperature and mole fraction on viscosity and thermal conductivity of water and ethanol mixture. In IOP Conference Series: Earth and Environmental Science; IOP Publishing, 2019; Vol. Vol. 227, p 042048.

[31] Gajardo-Parra N. F., Meneses L., Duarte A. R. C., Paiva A., Held C. (2022). Assessing
the influence of betaine-based natural deep eutectic systems on horseradish
peroxidase. ACS Sustainable Chem. Eng..

[32] Narita Y., Inouye K. (2013). Degradation kinetics
of chlorogenic acid at various
ph values and effects of ascorbic acid and epigallocatechin gallate
on its stability under alkaline conditions. J. Agric. Food Chem..

[33] Ruesgas-Ramón M., Suárez-Quiroz M. L., González-Ríos O., Baréa B., Cazals G., Figueroa-Espinoza M. C., Durand E. (2020). Biomolecules extraction from coffee and cocoa by-and
co-products using deep eutectic solvents. J.
Sci. Food Agric..

[34] Yue Y., Huang Q., Fu Y., Chang J. (2020). A quick selection of
natural deep eutectic solvents for the extraction of chlorogenic acid
from herba artemisiae scopariae. RSC Adv..

[35] Rojas-González A., Figueroa-Hernández C. Y., González-Rios O., Suárez-Quiroz M. L., González-Amaro R. M., Hernández-Estrada Z. J., Rayas-Duarte P. (2022). Coffee chlorogenic
acids incorporation for bioactivity enhancement of foods: A review. Molecules.

