# Supplementary material for: Extraction and Thermal Stability of Chlorogenic Acid from Green Coffee and Yerba Mate in Food-Based Solvents
Source: ACS Omega. 2026 Aug 10;11(33):49815–24. doi: 10.1021/acsomega.6c04214 (PMC13520052; doi:10.1021/acsomega.6c04214)
Supplement: Supplementary file 1 [file ao6c04214_si_001.pdf]

Extraction and thermal stability of chlorogenic acid from green coffee and yerba mate in food-based solvents

Monique Martins Strieder [a*], Giovanna Oliveira do Carmo [a], Érica Kaori Matsusaka Ijichi [a], Anna Giulia Correia Menino [a], Felipe Sanchez Bragagnolo [a], Mauricio Ariel Rostagno [a*]

[a] Faculdade de Ciências Aplicadas (FCA), Universidade Estadual de Campinas (UNICAMP), Rua Pedro Zaccaria, 1300, Limeira, SP-CEP 13484-350, Brazil

*Corresponding authors: mms@unicamp.br and rostagno@unicamp.br

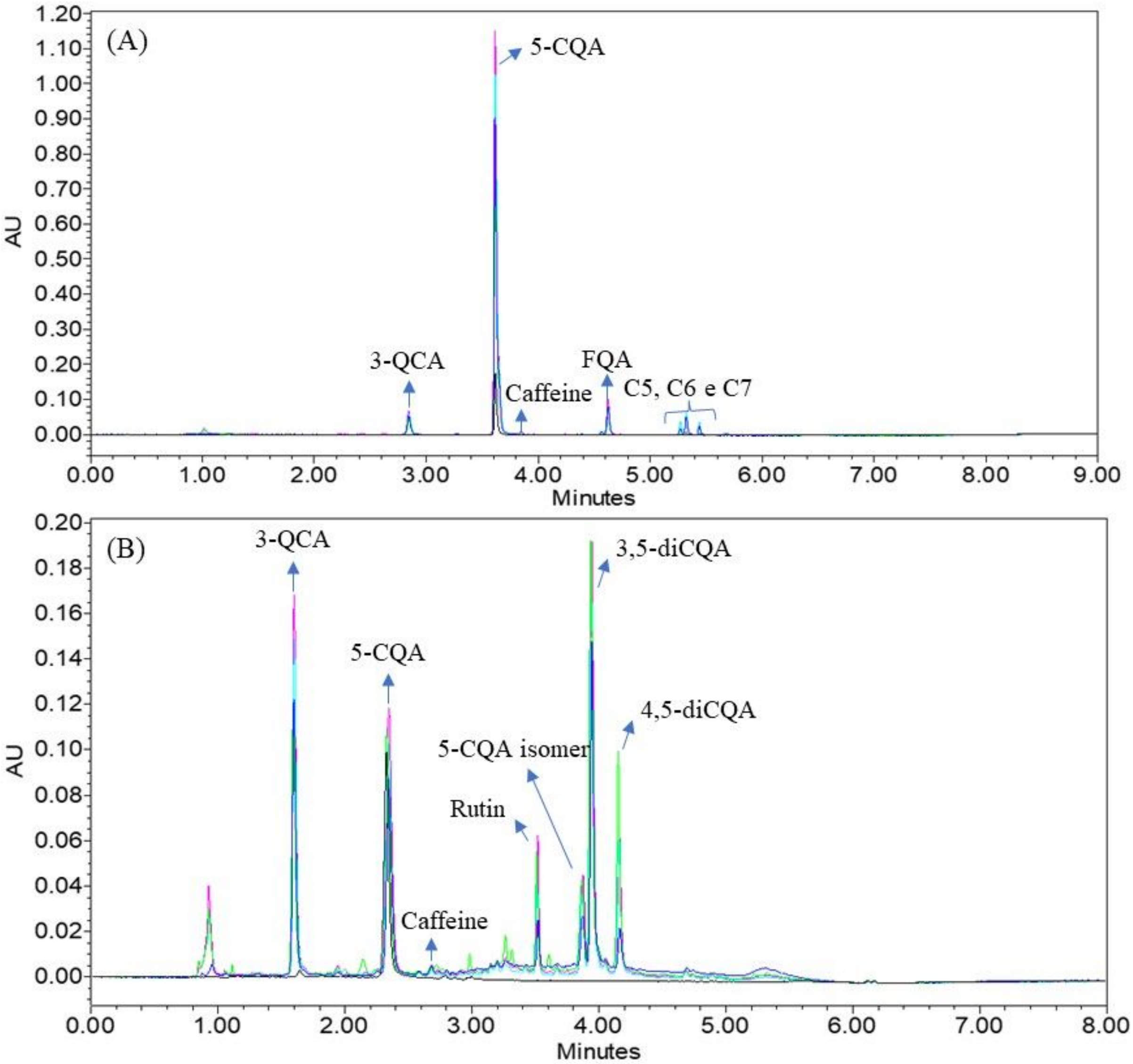

Figure S1. Chromatograms obtained at 350 nm for extracts of (A) green coffee and (B) yerba mate.
